# Annotations

**Published:** 1910-08-27

**Authors:** 


### Annotations.

### The late Sir Constantine Holman.

The death of Sir Constantine Holman comes as a surprise to his many friends who had come to look upon this venerable member of the profession as one to whom the gift of perpetual youth had been vouchsafed. For Constantine Holman was always, despite his eighty odd years of life, a hale and vigorous colleague whose exuberance of spirit and freshness of mind put to shame many a much younger member of the profession. An old Edinburgh student, the Scottish University gave him his M.D. in the early fifties, and at London he received his M.R.C.S., soon after starting in private practice. He rapidly proved himself an able clinician, and an embodiment of the best type of medical man. With these characteristics to help him, success was not long to seek, and Holman rapidly pushed his way to the front rank. He was a member of the Council of the Clinical and Obstetrical Societies, a Fellow of the Royal Society of Medicine, and a Vice-President of the British Medical Association, in which he took a great and eminently practical interest, having at one time been its treasurer and gold medallist. His work in connection with Epsom College, of which he was vice-president and honorary treasurer, made him known to many to whom his brilliant work as a clinician is comparatively little known. He took an immense interest in the work of the college, and the institution owes him a depth of gratitude for his efforts on its behalf. Foreign societies delighted to honour him by conferring upon him their honorary membership, and in 1904 his merits were suitably recognised by a knighthood. To his colleagues, and more especially to those in private practice, he was a sincere friend whose advice and assistance were always available. His death will be felt as a personal loss by many, and to Epsom scholars especially it will mean one which only they can sufficiently realise.

### The "Lady with the Lamp."

On Saturday last, with the drizzling rain falling just as it did on the memorable evening when she landed in the Crimea, Miss Florence Nightingale was borne to her last resting place in the little country churchyard at East Wellow, the spot she had herself selected with the wish, so characteristically expressed, that her burial should be devoid of pomp or ceremony. The nation that had learned to appreciate her services, that loved her as, it is no exaggeration to say, it has never loved a national heroine before, wished to accord her a fitting resting place in the Abbey, where so many great benefactors rest. But her own wish was peremptory, and after all it is a slight satisfaction, to those who hold that the "Lady with the Lamp" surpasses most of her contemporaries, to reflect that many of the greatest characters of the race sleep beyond Westminster's walls, and that more than one quiet country churchyard in England holds the mortal remains of men and women whose merits are recognised throughout the Empire. Last Saturday's funeral was in keeping with the unostentatious simplicity of Miss Nightingale's life, and the large throng of silent spectators who, despite the expressed wish of her executors that the ceremony should be strictly private, gathered round the churchyard, showed how deeply her death was felt. A pile of wreaths and floral tributes—conspicuous among them being the contributions from hospitals and nursing staffs, and among these again the special wreaths sent by the institutions with which the deceased had been particularly associated during her lifetime—was an added proof of widespread regret and sympathy. The simple memorial service in St. Paul's, attended by a large concourse of friends and others who, although they could not claim to have known Miss Nightingale personally, were yet sufficiently appreciative of her life's work to render homage to her memory, was impressive through its very simplicity, while the memorial services in other churches were equally well attended. Though her last wishes have debarred the nation from giving such expression to its feeling of profound admiration and respect as it would have liked to give, there is yet an opportunity for crystallising that feeling into a tangible memorial to her greatness, and we have no doubt that in the near future means will be found to perpetuate her memory in a manner befitting the simple grandeur of her life and the unselfish ideals she aimed at.

### A Health Caravan.

The christening of the Aurora caravan in Regent's Park on Saturday last marks a praiseworthy commencement in a peculiar line of personal service that ought to have the cordial approval of all members of the medical profession. The caravan has been equipped by the Women's Imperial Health Association, and will travel about in country districts in charge of competent lecturers furnished by this society with the object of expounding simple rules of hygiene to the public. We have been favoured with copies of the leaflets which the Association intends to distribute, and we have found them excellent in every way. They set forth, in simple, straightforward language, easily understood by everyone who is capable of reading, the equally simple rules of health which, if they were only followed by every man, woman, and child in this or in any other country, would lead to an inevitable improvement in national health. It is, indeed, gratifying to find that the Association has no axes to grind, no fads to ventilate, no isms or antis to preach. We would suggest to the Association the desirability of forwarding a supply of its literature to every doctor, clergyman, and teacher in the country, with a request to aid in its distribution and to help the work that the caravan lecturers are doing. One of the pamphlets, entitled "The Ten Commandments," is particularly suitable for free distribution. We are looking forward to the first report of the Association in connection with the novel tour, and meanwhile wish the Aurora and its staff a prosperous and successful pioneering career.

